# RETRACTION: The Mechanism of the Yigutang‐Mediated P13K/AKT/GSK‐3β Signal Pathway to Regulate Osteogenic Differentiation of Bone Marrow Stromal Stem Cells to Treat Osteoporosis

**DOI:** 10.1155/ecam/9769826

**Published:** 2026-10-22

**Authors:** Evidence-Based Complementary and Alternative Medicine

RETRACTION: N. Li, Y. Gong, “The Mechanism of the Yigutang‐Mediated P13K/AKT/GSK‐3β Signal Pathway to Regulate Osteogenic Differentiation of Bone Marrow Stromal Stem Cells to Treat Osteoporosis,” *Evidence-Based Complementary and Alternative Medicine*, 2021, 6699781, https://doi.org/10.1155/2021/6699781.

The above article, published online on 18 June 2021 in Wiley Online Library (https://wileyonlinelibrary.com), has been retracted by John Wiley & Sons Ltd. The retraction has been agreed due to concerns with the western blots shown in Figures 5–8. Specifically, the authors originally informed us that the current blots for Figures 5–8 were incorrect and from a different experiment. On investigation, it was noted that the revised blots used GAPDH as the loading control instead of ß‐actin.

Following an assessment of the concerns raised, together with the author’s response and associated raw data, the Integrity Assurance team has concluded that the results and conclusions can no longer be considered reliable. The article is therefore retracted.

The authors disagree with the retraction.

